# Foot and ankle service adaptation in response to COVID-19 and beyond

**DOI:** 10.1016/j.amsu.2020.04.023

**Published:** 2020-04-28

**Authors:** I. Feeley, T. McAleese, K. Clesham, D. Moloney, G. Crozier-Shaw, A. Hughes, T. Bayer

**Affiliations:** a Department of Trauma and Orthopaedics, Midlands Regional Hospital Tullamore, Tullamore, Ireland; b National University of Ireland Galway, Co Galway, Ireland

**Keywords:** Orthopaedic service adaptation, Inter-disciplinary teams, Healthcare technology, Public health, COVID-19

## Abstract

The disruption to healthcare provision as a result of the COVID-19 pandemic has compelled us to streamline healthcare delivery. This has given us an opportunity to implement healthcare technology, reform inter-disciplinary collaboration and ultimately enhance patient care. We discuss some of the advances made by the foot and ankle department at our hospital. These innovations have broad applicability and will hopefully ignite discussion amoung a number of healthcare teams about improving the future care of their patients.

The COVID-19 pandemic has caused unparalleled disruption to the provision of healthcare. The repercussions of this pandemic extend beyond the direct effect of the virus itself as other services contemporaneously adapt to this new challenge. John F. Kennedy may have infamously misinterpreted the Chinese symbol for crisis simultaneously representing danger and opportunity but the sentiment perseveres and it is well recognised in the field of economics that crises do induce reform [1]. The work environment in which orthopaedic surgeons now practice has shifted rapidly and is ever-evolving. We propose this will serve as a catalyst for changing our clinical practice in the future in a way that will ultimately improve patient care. We examine the changes that have been introduced to streamline the foot and ankle service in our hospital in light of the current crisis and the underlying evidence for these changes.

The widespread introduction of instant messaging applications (IMAs) has improved internal, synchronous communications within healthcare teams compared to the prior paging and bleep systems [2]. Whatsapp is the most popular of these applications worldwide, although there are limited guidelines regarding its use for sensitive patient data, compliance with General Data Protection Regulation (GDPR) and concerns have been raised regarding the security of its end-to-end encryption system [3,4]. Our service recently established a formal referral pathway between the emergency department and the orthopaedic service using Siilo, an IMA marketed as a GDPR-compliant alternative to Whatsapp [5]. Referrals containing a brief history and relevant imaging are sent into a Siilo group, which includes both orthopaedic and emergency medicine consultants. This multimedia evolution from the preceding pager system allows rapid, consultant lead decision-making, minimising the time between referral and a final management decision and serves as a quality assurance mechanism for resident decisions documented in the group. It also facilitates immediate input from sub-specialist interest groups when required without recourse for attending an additional outpatient appointment. Another positive reverberation has been the reduced congestion at emergency department workstations and computers due to an overall decrease in human traffic. Use of digital referral pathways are supported by a strong evidence base in a number of different specialties [6,7]. They markedly reduce response times to referrals [8], and allow specialist treatment advice be relayed to remote sites [9].

Our unit provides orthopaedic services to a predominantly rural catchment area of over 400,000 people. It was an early advocate of telemedicine and virtual fracture clinics (VFC) for referrals from surrounding institutions with no dedicated orthopaedic department [10]. Our current healthcare environment has seen us expand our VFC criteria to include all new referrals from both outside and within our institution and to include follow-up for patients who previously would have been seen at in-hospital clinics. Should imaging be necessary this can be arranged at a local unit and reviewed remotely on the national integrated medical imaging system (NIMIS). Our new criteria have reduced clinic attendance from a mean of 68 to fewer than 10 patients in the 4 weeks since stringent national measures were introduced to “flatten the curve” of the COVID-19 pandemic.

Our weekly trauma meeting has been reconstituted to videoconference form using “Zoom”. The presentation and imaging is screen-shared and discussed remotely between off-site members of the department while simultaneously being projected in our conference room. It is only attended in person by the trauma team on call that day that conducts the proceedings. Another teleconference involving all the consultant staff takes place at the end of each working day to facilitate accurate handover and ongoing discussions regarding policies and adaptations to the ever-changing situation.

Telemedicine has been utilised since the early 1990s across all surgical specialties initially being pioneered in areas with low population densities [11]. It has demonstrated excellent patient satisfaction scores for initial assessments [12] and post-operative follow up and results in significant cost savings [13,14] In-person patient care should be recognised as an important part of the provider-patient relationship but we suggest continuing broad inclusion criteria for virtual clinics, even once the current societal restrictions have been lifted, is beneficial for patients and doctors [15].

The standard post-operative management after operative foot and ankle procedures in our unit was to allow patients to weight bear as tolerated with early return to unprotected mobilization [16]. We conduct outpatient follow-up at 1 week for compression bandage change, 2 weeks for compression bandage change and suture removal and 6 weeks to perform a check X-ray and remove the controlled ankle movement (CAM) boot if appropriate. Further follow-up is arranged as needed. However, on the basis of a number of recent articles we have now curtailed routine follow-up X-rays after operative fixation of ankle fractures. Van Gerven et al. found only 1.2% of 1174 radiographs booked routinely for 528 patients post ankle ORIF resulted in changes to management [17]. Instead, we use focused imaging as indicated by patient symptoms or signs. The need for routine follow-up of wounds is maintained and Ovaska et al. found that only 37% of wound complications presented with an unscheduled visit. [21] However, our practice of only using removable CAM splints mitigates cast-related complications and will allow virtual or public health nurse wound-assessment. We are in the process of setting-up remote wound assessment as proposed in “The journal of the American College of Surgeons” in 2018 who outlined a specific protocol for vascular surgery patients who took photographs and dressed wounds at home [18].

We have re-designed our rehabilitation and physiotherapy protocols. Previously, we had routinely referred most operative and non-operative foot and ankle injuries for supervised physiotherapy. We have now created a new website (www.orthotac.ie) dedicated to uploading videos of exercises performed by qualified physiotherapists and access to patient information leaflets (PIL) for common injuries. Our review of the evidence suggests that a supervised rehabilitation programme confers no additional benefits for both operative and non-operative groups after immobilisation for isolated ankle fractures when compared to physiotherapy advise alone [19]. This is a modification of tele-rehabilitation, which has shown much promise in hip and knee arthroplasty [20].

## Provenance and peer review

Not commissioned, editor reviewed.

## Ethical approval

Ethical approval was not required and patient-identifying information was not presented in this report.

## Sources of funding

This research did not receive any specific grant from funding agencies in the public, commercial, or not-for-profit sectors.

## Author contribution

**Iain Feeley:** Conceptualisation, writing-original draft.

**Timothy McAleese:** Conceptualisation, software, writing – original draft.

**Kevin Clesham:** Conceptualisation and design, Project administration, writing – review and editing.

**Darren Moloney:** Conceptualisation, investigation, writing – review and editing.

**Geoff Crozier-Shaw:** Conceptualisation, administration, resources, formal analysis, writing – review and editing.

**Andrew Hughes:** Conceptualisation, administration, validation, visualisation, writing – review and editing.

**Thomas Bayer:** Supervision, conceptualisation, validation, writing – review and editing.

All authors read and approved the final manuscript.

## Registration of research studies

This is not applicable due to the nature of this “Commentary”.

## Guarantor

Mr Thomas Bayer is a consultant orthopaedic surgeon and the senior author in this paper. As guarantor he is responsible for the work and/or the conduct of the commentary and controlled the decision to publish.

## Declaration of competing interest

The authors have no conflicts of interest to declare.
